# Longitudinal and transverse muscle stiffness change differently with knee osteoarthritis and do not align with stiffness sensation

**DOI:** 10.3389/fphys.2025.1593851

**Published:** 2025-05-27

**Authors:** Angela V. Dieterich, Katrin Skerl, Filip Paskali, Leonardo Gizzi, Mehrin Azan, Gabriela F. Carvalho, Matthias Kohl, Andreas Haueise

**Affiliations:** ^1^ Ultrasound Lab, Department of Physiotherapy, Faculty of Health, Medical, and Life Sciences, Furtwangen University, Freiburg, Germany; ^2^ Institute of Technical Medicine, Faculty of Health, Medical, and Life Sciences, Furtwangen University, Furtwangen, Germany; ^3^ Institute of Precision Medicine, Faculty of Health, Medical, and Life Sciences, Furtwangen University, Villingen-Schwenningen, Germany; ^4^ Fraunhofer Institute for Production Engineering and Automation, Department of Biomechatronics, Stuttgart, Germany; ^5^ Institute for Modelling and Simulation of Biomechanical Systems IMSB, University of Stuttgart, Stuttgart, Germany; ^6^ Department of Sport and Sport Science, University of Freiburg, Freiburg, Germany

**Keywords:** shear wave elastography, muscle, stiffness, knee osteoarthritis, anisotropy, age, patient reported outcome measures

## Abstract

Knee osteoarthritis (OA) is a prevalent joint condition associated with disability, pain, and stiffness in the muscles surrounding the knee. Myofascial and massage techniques are employed to alleviate these symptoms. Unclear is whether muscle stiffness is measurably increased in the painful muscles, and how measured muscle stiffness relates to perceived stiffness, pain, and functional impairment. Given muscle anisotropy, stiffness changes may occur in the longitudinal plane parallel to muscle fibers or perpendicularly in the transverse plane. Shear wave velocity (SWV) was measured in both scanning planes in 21 individuals with diagnosed knee OA, 21 sex- and age-matched controls, and 20 *young controls*, focusing on the gastrocnemius medialis and vastus lateralis muscles under four conditions: (a) resting state, (b) holding the shank against gravity, (c) double-leg stance, and (d) single-leg stance. Median stiffness measures, the ratio of longitudinal-to-transverse stiffness, and the ratio of single-leg stance-to-baseline stiffness were compared between groups using the Kruskal- Wallis and Pairwise Asymptotic Wilcoxon rank sum tests. Correlations with the Knee Injury and Osteoarthritis Outcome Score and the Tampa Scale of Kinesiophobia were examined. Longitudinal stiffness of the gastrocnemius medialis muscle was significantly lower in the OA group at double-leg (P = 0.033) and single-leg stance (P = 0.019), with tendencies toward lower median stiffness in both muscles across most tasks. Transverse stiffness of the vastus lateralis muscle was significantly higher in the OA group at baseline (P = 0.027), with tendencies toward higher median stiffness in both muscles across most tasks. Significant moderate to excellent correlations support the clinical relevance of both longitudinal and transverse stiffness measurements. Measured and perceived stiffness were not correlated. Study results suggest that knee OA may differentially affect muscle stiffness in the longitudinal and transverse planes and that transverse stiffness measures may have clinical relevance.

## 1 Introduction

Osteoarthritis (OA) of the knee is the most prevalent form of osteoarthritis (Long et al., 2022) characterized by progressive limitations on ambulatory function and joint mobility, significantly impacting the individual’s quality of life (Driban et al., 2020). The perception of joint stiffness is one of the major clinical symptoms of osteoarthritis (Bastick et al., 2016). Patients with knee OA commonly report pain and stiffness in the muscles surrounding the knee joint (Teo et al., 2019; NICE, 2023). Physiotherapy is the first-line treatment recommended for alleviating symptoms and functional impairment (Gibbs et al., 2023). In response to patients’ complaints and based on the assumption of objectively increased muscle stiffness, interventions such as massage (Bahns and Kopkow, 2023) or myofascial techniques (Dor and Kalichman, 2017) are often employed aiming to reduce muscle stiffness. However, it remains unclear whether muscle stiffness is objectively increased in the affected muscles, and how measured muscle stiffness relates to perceived stiffness, pain, and functional impairment.

Ultrasound shear wave elastography is the most frequently used modality to non-invasively estimate tissue stiffness, with muscle shear wave velocity (SWV) or the related shear modulus serving as the primary measures of stiffness (Davis et al., 2019; Wang et al., 2019). To date, two studies explored muscle stiffness using shear wave elastography around the knee joint in patients with knee OA compared to healthy individuals (Gökşen et al., 2021; Li et al., 2021). Gökşen et al. found no significant difference in SVV of the rectus femoris and the biceps femoris muscles between individuals with and without knee OA (Gökşen et al., 2021) while Li et al. reported an increased shear modulus in all three hamstring muscles (Li et al., 2021). In both studies blinding was not reported and manually selected small regions of muscle tissue were measured, introducing a potential risk of bias and raising concerns about representative measurements (Haueise et al., 2023). Additionally, it remains unclear whether the measured muscles were perceived as stiff by the patients. Thus, current literature does not answer the question whether subjective and objective muscle stiffness correspond. Moreover, although patients with musculoskeletal conditions frequently report sensations of tense or stiff muscles, evidence for objectively increased muscle stiffness is conflicting (Haueise et al., 2023). All studies included in the aforementioned review measured muscle stiffness only along muscle fiber direction, which is the standard procedure (Săftoiu et al., 2019). Previous research suggests that large samples (Kolding et al., 2018; Hvedstrup et al., 2020; Li et al., 2023) and eventually the summation of stiffness measures across different muscle parts (Li et al., 2023) may be necessary to detect measurably increased muscle stiffness in pain conditions. This contrasts with the prevailing clinical perception of palpably increased muscle stiffness. The question arises of whether the well-established longitudinal shear wave elastography measurements capture the phenomenon underlying perceived muscle stiffness sufficiently.

Muscle tissue is anisotropic, meaning its stiffness differs between the longitudinal (parallel to the direction of muscle fibers) and the transverse (cross-sectional) planes (Green et al., 2024; Gennisson et al., 2010; Ngo et al., 2024). Longitudinal muscle stiffness reflects both active and passive tension, increasing with muscle activation and stretch (Hug et al., 2015). Transverse muscle stiffness is thought to represent the mechanical properties in cross-sectional direction. Previous studies have indicated that muscle stiffness is lower in the transverse plane and is less influenced by muscle activation compared to longitudinal measurements (Gennisson et al., 2010; Eby et al., 2013; Ngo et al., 2024). Muscle palpation and massage techniques are typically applied transversely to the direction of the muscle fibers (Kolster, 2016). This traditional approach may reflect implicit knowledge and experience of altered transverse muscle properties. To our knowledge, transverse muscle stiffness has not been measured in individuals with knee OA.

The mechanical properties of muscles change with age (Kragstrup et al., 2011; Lieber and Ward, 2013). To distinguish changes of muscle stiffness due to ageing from those associated with pain in OA, two control groups were included, an age- and sex-matched group to isolate OA-specific effects, and a younger sex-matched group to capture age-related changes. The primary aim of the current study was to compare SWV of the most painful muscles around the knee joint in both longitudinal and transverse planes across three groups 1) individuals with diagnosed knee OA, 2) age- and sex-matched control participants, and 3) sex-matched young participants under both resting and active conditions. A previous study questions a relationship between perceived and measured muscle stiffness (Akagi and Kusama, 2015). To examine this and the clinical relevance of the stiffness measures, the secondary aim was to investigate the relationships between measured muscle stiffness and clinical symptoms including pain, stiffness, knee joint function, and kinesiophobia.

## 2 Methods

This cross-sectional observational study was planned and conducted according to the STROBE-checklist for cross-sectional studies (von Elm et al., 2007). Recruitment and enrolment of participants were in accordance with the Declaration of Helsinki. Ethical approval was obtained from the Ethics Committee of the University of Stuttgart (No 21–012) and each participant provided informed consent prior to participation.

### 2.1 Participants

Participants aged 45–68 years, either diagnosed with knee osteoarthritis or free of lower limb conditions and discomfort, were recruited through university posters, local newspaper advertisements, and a digital neighborhood platform. Eligibility was assessed via telephone screening based on the following inclusion and exclusion criteria. The group of participants with knee OA (labelled *OA*) included individuals with a medical diagnosis of knee OA irrespective the OA stage, and painful muscles around the knee joint for at least 6 months. Both healthy control groups comprised individuals without lower limb diagnoses or pain lasting longer than a week within the past 2 years, and were labelled *old control* and *young control* to emphasize the age contrast, not to judge age. The age-matched control group included healthy individuals matched for age and sex, whereas the *young control* group included sex-matched healthy individuals aged 18–30 years. Exclusion criteria for all three groups included systemic diseases affecting muscle performance (e.g. rheumatic diseases, COPD), a history of knee or hip trauma (e.g. ACL rupture), intake of medication potentially affecting muscle tone (e.g. muscle relaxants), and skin lesions at the lower limb. An *a priori* power analysis was conducted using G*Power (Version 3.1.9.7), based on a cross-sectional study including four groups (Alfuraih et al., 2020). The effect size (f = 0.48) was calculated in G*Power based on the vastus lateralis SWV between the healthy and active rheumatoid arthritis groups, as these yielded the largest differences. As G*Power does not support the Kruskal–Wallis test, a one-way ANOVA was used as its parametric equivalent. With α = 0.05 and a power (1 – β) = 0.80, the required sample size was n = 45 for group comparisons and n = 38 for pairwise Wilcoxon tests. The participants of the OA group were recruited first to allow for matching of the control groups. Demographic information and self-reported measures were collected from all participants using the German versions of the “Knee Injury and Osteoarthritis Outcome Score” (KOOS) (Kessler et al., 2003) and the “Tampa Scale of Kinesiophobia” (TSK) questionnaires (Rusu et al., 2014). The KOOS questionnaire includes subscores for pain, functional limitations, and symptoms, including two items (S6, S7) specifically assessing the intensity of perceived stiffness.

### 2.2 Procedure

Muscle stiffness was estimated using an Aixplorer Ultimate system (V.12, Supersonic Imagine, Aix-en-Provence, France) equipped with a linear transducer (SL15-4) with a 5 cm footprint. The system was configured for shear wave elastography, MSK preset with 90% gain, depth 2.0–2.5 cm, focus zone centered, sampling frequency 1.2–2.0 Hz, dynamic range 72 dB, a maximal measurement range of <10 m/s, low persistence, a standard setting between resolution and penetration, and 50% opacity.

**FIGURE 1:**
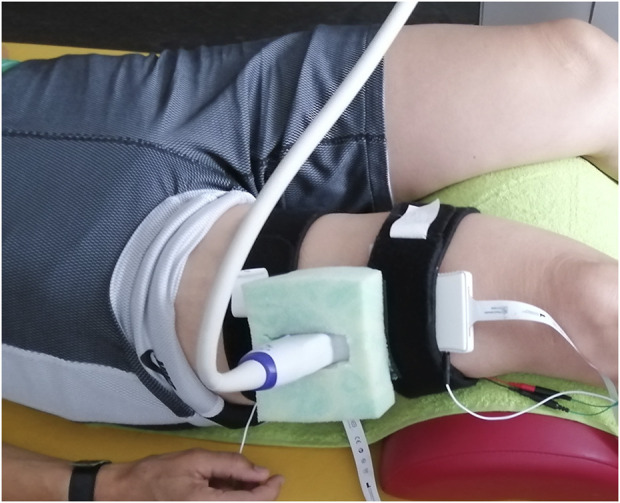
The ultrasound transducer was hosted in a custom-cut foam block, here for recording the M. vastus lateralis.

After completing demographic information and questionnaires, participants were prepared for data collection. Individuals with knee OA were asked to point out and rank their two most painful muscles. The most painful muscle was then scanned using B-mode ultrasound to determine and mark the direction of the muscle fibers, a longitudinal and a transverse scanning location. The locations were chosen on top of the muscle belly, approximately in accordance with the SENIAM recommendations (Hermens et al., 2000). Adhesive foam tape was used to mark and frame the transducer locations to ensure consistency for repeated measurements (Dieterich et al., 2020). To standardize the potential influence of previous activity (Proske and Gandevia, 2012), the prepared participants walked for 5 min at comfortable pace on a treadmill. Data collection started with participants positioned supine or prone on a plinth, depending on their most painful muscle. The ultrasound probe was hosted in a custom-cut foam block, covered with sufficient ultrasound gel, and secured to the leg with hook-and-loop straps (Figure 1). The elastogram was configured to cover the maximum visualizable area of the target muscle, positioned at a depth of 1.2–1.5 cm just below the superficial fascia, to optimize area-based visualization of muscle tissue. SWV of the most painful muscle was recorded at four different conditions, 1) *baseline*, i.e., resting state, 2) holding the shank against gravity (*lift shank*), 3) *double-leg stance* and 4) *single-leg stance* (Table 1). Measurements were taken in longitudinal and in transverse transducer orientation. Trials were recorded as 30-s video clips except *single-leg stance* (20 s) to prevent exacerbation of pain. Dependent on recording duration and depth, each clip included 24–54 elastograms (images). All conditions except baseline were repeated three times. After recording SWV in the most painful muscle, measurements were taken from the second most painful muscle only during the more challenging tasks *double-leg* and *single-leg stance*. The same muscles considered as the first and second most painful muscles by a participant of the *OA* group were measured in a sex- and age matched participant from the *old control* group and a sex-matched participant from the *young control* group. Data were stored without any indication of group allocation.

**TABLE 1:** Description of the recording conditions. All conditions were recorded in longitudinal and in transverse transducer orientation. Depending on the most painful muscle, participants were positioned supine or prone on the plinth.

Trial label	Description	Verbal instruction
*Baseline*	resting state, depending on recorded muscle supine or prone with a roll under the knees or ankle joints (Figure 1), 30 s (appr. 48 elastograms)	“Stay as relaxed as possible”
*Lift shank*	active knee extension/flexion against gravity with a roll under the knee joint 30 s voluntary isometric contraction in prone/supine, only weight of the shank	“Extend/Flex you knee until your shank has no contact to the plinth and hold the position for 30 s”
*Double-leg stance*	30 s bipedal standing, feet hip-width apart, eyes closed	“Close your eyes, do not talk and focus on your breath”
*Single-leg stance*	20 s shifting the weight to the recorded leg as far as pain intensity allows, with toe contact of the unloaded leg	“Shift your weight to the left/right leg until you reach your pain threshold. Keep slight contact with the other foot to support your balance.”

### 2.3 Data processing

**FIGURE 2:**
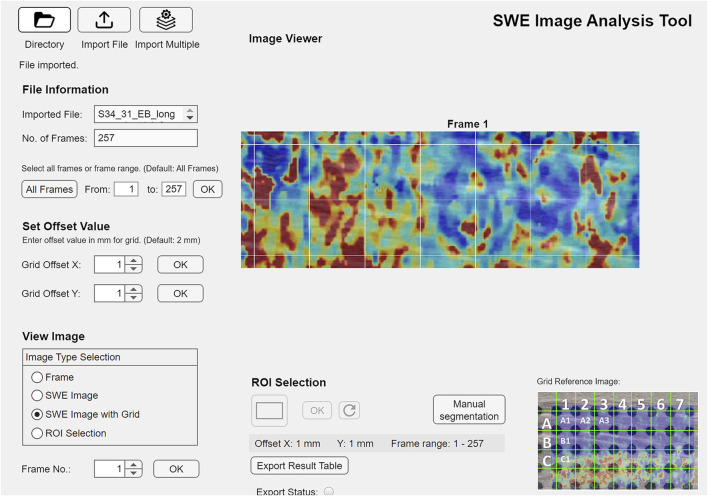
GUI (graphical user interface) of the custom-programmed script for SWE data processing, example from the *young control* group, M. gastrocnemius medialis, single-leg stance, longitudinal transducer orientation.

Elastography clips were exported as DICOM files and computer-analyzed using a custom script (MATLAB R2021b, The MathWorks, Natick, MA, United States) modified from Skerl et al. (Skerl et al., 2017) to extract stiffness measures from the complete elastograms within the clips. The SWE clips comprised greyscale B-mode ultrasound data at 11 Hz and stiffness data (elastography) at 1.2–2.0 Hz. Each clip contained 200–750 greyscale images with 24–54 superimposed elastograms. The elastogram’s position within the greyscale images was automatically detected and the elastogram’s upper 1 mm was discarded. The elastogram was segmented into 14 small (4 mm × 4 mm) squares (Figure 2) from which stiffness was extracted separately. For each square in each frame of a clip, the following data were extracted: maximum, mean and median SWV, standard deviation and interquartile range, percentage of colored pixels (representing stiffness information), and the number and percentage of pixels with a SWV >9.9 m/s (suggesting saturated measurements). As a measure of imaging quality control, the number of pixels containing colored elastography values was calculated for each square in each frame. Frames containing less than 50% colored pixels were excluded from further analyses. If more than 50% of the squares were excluded, the elastogram was discarded. Extracted data were exported for each clip separately for the statistical analyses. Group allocation was not disclosed prior to the statistical analysis.

### 2.4 Statistical analysis

Demographic data, the sum of the KOOS S6 and S7 items asking for stiffness sensations, and the results of the KOOS and TSK questionnaires were described per group by mean and standard deviation. Statistical analysis of the stiffness data was performed using R (Team, 2023) with the following packages: tidyr (Wickham et al., 2023b) and dplyr (Wickham et al., 2023a) for data handling, MKinfer (Kohl, 2023) and SPSS (V.29, IBM corporation) for statistical analysis, and ggplot2 (Wickham, 2016) and patchwork (Pedersen, 2023) for data visualization.

The extracted data included median stiffness values from squares (1–7) in two rows (A; B; Figure 2) over 20- or 30-s recording time. Measurements of SWV from the first and second most painful muscles were combined for analysis. Longitudinal and transverse measurements were evaluated separately for each muscle. Given that muscle function involves three-dimensional changes in muscle shape (Azizi et al., 2017), mechanical alteration in one plane likely relates to the mechanical properties in other planes. Consequently, the ratio of longitudinal to transverse SWV was calculated as a two-dimensional indicator of longitudinal muscle tension relative to transverse elasticity. Additionally, the ratio of SWV at baseline to that during single-leg stance was computed as an indicator of stiffness change with a high activation level. Stiffness measures and stiffness ratios were analyzed by group and muscle.

Reliability of the stiffness measures between repeated recordings was estimated using the ICC(3,3) in SPSS (IBM, Version 29, reliability analysis two-way mixed absolute agreement) and the standard error of measurement (SEM). The Shapiro-Wilk test demonstrated skewed data distributions for most testing conditions. Therefore, muscle stiffness and stiffness ratios were described by median and interquartile range (IQR). Group differences were examined using the Kruskal–Wallis Rank Sum test. Significance was set to P < 0.05. For significant group differences, Pairwise Asymptotic Wilcoxon Rank Sum Tests were used to identify the different groups. Within the knee OA group, the Spearman correlation coefficient *ρ* (rho) was used to assess the relationships between stiffness measures, stiffness sensations, the KOOS domains, the TSK scores, age, and BMI. Correlation coefficients *ρ* > 0.75 were judged good to excellent, *ρ* between 0.50 and 0.75 moderate to good, and *ρ* between 0.25 and 0.50 fair (Portney and Watkins, 2000).

## 3 Results

Sixty-two participants took part in the study (Table 2), resulting in 1240 trials of the most painful muscle and 740 trials of the second most painful muscle, which was only recorded during standing activities. In the OA group, the most painful muscle was identified as the vastus lateralis in 11 participants, the gastrocnemius medialis in 7 participants, the vastus medialis in 2 participants, and biceps femoris in 1 participant. For the second most painful muscle, the vastus lateralis was selected by 4 participants, the gastrocnemius medialis by 11 participants, the vastus medialis by 3 participants, and the biceps femoris by 3 participants. Due to the limited number of measurements of the vastus medialis and biceps femoris muscles, the presented results focus on the vastus lateralis and gastrocnemius medialis muscles. Data inclusion following the quality control described in 2.3 was on average 85% for longitudinal scanning and 74% for transverse scanning, varying by muscle. The highest data inclusion was observed for the gastrocnemius medialis muscle in the longitudinal plane (98%), while the lowest was for the vastus lateralis muscle in the transverse plane (64%). The reliability of stiffness measurements was higher in longitudinal than in transverse measurements (Table 3).

**TABLE 2:** Demographic description and patient reported outcome measures (KOOS and Tampa scale of kinesiophobia) of the three groups; mean ± SD. Perceived stiffness = KOOS items S6 + S7.

	Knee OA group	Old control group	Young control group
N (female:male)	21 (16:5)	21 (16:5)	20 (15:5)
Age (years)	57.4 ± 6.3	58.0 ± 5.6	22.1 ± 2.8
Height (cm)	171.7 ± 6.7	172.0 ± 6.9	171.7 ± 7.3
Weight (kg)	74.7 ± 12.7	71.6 ± 11.7	65.7 ± 9.4
BMI	25.3 ± 3.9	24.2 ± 3.7	22.2 ± 2.3
KOOS Perceived stiffness - Pain - Symptoms - ADL - Sport - QOL	3.3 ± 1.8 55.4 ± 13.8 55.9 ± 18.0 70.7 ± 16.6 28.5 ± 22.6 34.2 ± 18.3	0.1 ± 0.4 97.8 ± 4.5 96.8 ± 3.6 99.3 ± 1.7 96.4 ± 8.2 97.3 ± 7.5	0.2 ± 0.9 97.6 ± 6.1 95.5 ± 6.4 99.5 ± 1.3 98.9 ± 3.5 96.9 ± 9.6
Tampa Scale of Kinesiophobia	21.2 ± 5.5	17.9 ± 5.4	15.7 ± 3.6

Abbreviations: ADL, activities of daily living; BMI, body mass index; KOOS, Knee osteoarthritis outcome score; OA, osteoarthritis; QOL, quality of life.

**TABLE 3:** Reliability (ICC(3,3) of median stiffness measures in the recording conditions that were repeated three-times, mean (95% confidence intervals) over squares.

Muscle scanning direction	Lift shank	Double-leg stance	Single-leg stance
M. gastrocnemius medialis longitudinal	0.833 (0.619–0.937) SEM 0.129 m/s	0.981 (0.969–0.988) SEM 0.247 m/s	0.990 (0.983–0.994) SEM 0.243 m/s
M. vastus lateralis longitudinal	0.989 (0.978–0.995) SEM 0.077 m/s	0.956 (0.917–0.977) SEM 0.118 m/s	0.959 (0.928–0.977) SEM 0.116 m/s
M. gastrocnemius medialis transverse	0.833 (0.619–0.937) SEM 0.129 m/s	0.981 (0.969–0.988) SEM 0.247 m/s	0.990 (0.983–0.994) SEM 0.243 m/s
M. vastus lateralis transverse	0.833 (0.619–0.937) SEM 0.129 m/s	0.981 (0.969–0.988) SEM 0.247 m/s	0.990 (0.983–0.994) SEM 0.243 m/s

Abbreviations: ICC intraclass correlation coefficient; *M. musculus*; SEM standard error of measurement.

### 3.1 Longitudinal muscle stiffness

**FIGURE 3:**
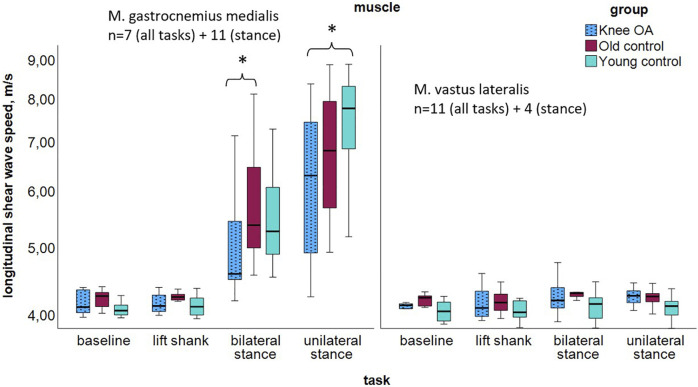
Longitudinal shear wave velocity of the gastrocnemius medialis and vastus lateralis muscles per group at resting state and tasks of increasing activity; logarithmic scaling of the x-axis to enhance the visibility in the lower range. *P < 0.05. The high gastrocnemius medialis stiffness at stance is likely due to the stretch over two joints. Abbreviations: M., muscle; n, number of participants per group; OA, osteoarthritis.

Longitudinal measurements in both muscles indicated tendencies toward lower median SWV in the *knee OA* group (Figure 3; Table 4), with significance in the Kruskal Wallis Rank Sum Test observed for the gastrocnemius medialis muscle at *double-leg stance*, P = 0.033, and at *single-leg stance*, P = 0.019, groupwise comparisons see Table 4. At *baseline* and *lift shank*, the *young control* group demonstrated the lowest and the *old control* group the highest longitudinal stiffness. In the *knee OA* group, median longitudinal stiffness at *baseline* and *lift shank* was similar to the *young control* group.

**TABLE 4:** Longitudinal shear wave velocity of the gastrocnemius medialis and vastus lateralis muscles per group at resting state and tasks of increasing activity, P values for group differences.

Task & muscle	Knee OA group (1) median (IQR)	Old control group (2) median (IQR)	Young control group (3) median (IQR)	P Value Kruskal Wallis test	P Value pairwise comparisons
Baseline GM	4.098 (0.39) m/s	4.254 (0.30) m/s	4.050 (0.17) m/s	0.217	
Lift shank GM	4.114 (0.27) m/s	4.240 (0.11) m/s	4.103 (0.34) m/s	0.528	
Double-leg stance GM	4.580 (1.23) m/s	5.373 (1.66) m/s	5.264 (1.31) m/s	0.033*	(1) vs. (2) 0.019* (1) vs. (3) 0.041*
Single-leg stance GM	6.292 (2.70) m/s	6.803 (2.34) m/s	7.754 (2.21) m/s	0.019*	(1) vs. (3) 0.008*
Baseline VL	4.122 (0.09) m/s	4.229 (0.16) m/s	4.040 (0.30) m/s	0.126	
Lift shank VL	4.086 (0.54) m/s	4.162 (0.28) m/s	4.027 (0.24) m/s	0.418	
Double-leg stance VL	4.192 (0.30) m/s	4.294 (0.15) m/s	4.142 (0.32) m/s	0.169	
Single-leg stance VL	4.257 (0.19) m/s	4.248 (0.15) m/s	4.112 (0.22) m/s	0.065	

Abbreviations: GM, gastrocnemius medialis muscle; IQR, interquartile range; OA, osteoarthritis; VL, vastus lateralis muscle; vs., *versus*; *P < 0.05.

### 3.2 Transverse muscle stiffness

**FIGURE 4:**
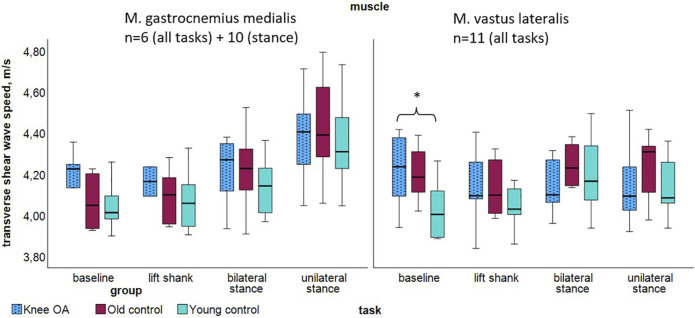
Transverse shear wave velocity of the gastrocnemius medialis and vastus lateralis muscles per group at resting state and tasks of increasing activity. *P < 0.05. Abbreviations: M., muscle; n, number of participants per group; OA, osteoarthritis.

Transverse measurements demonstrated tendencies toward higher median SWV in the knee OA group acoss most tasks (Figure 4; Table 5), with significance observed in the Kruskal Wallis Rank Sum Test for the vastus lateralis muscle, P = 0.026. In the *knee OA* group, transverse SWV showed minimal or no increase with rising activity levels. At *baseline* and *lift shank*, the *OA* group demonstrated the highest and the *young control* group the lowest transverse stiffness.

**TABLE 5:** Transverse shear wave velocity of the gastrocnemius medialis and vastus lateralis muscles per task and group, P values for group differences.

Task & muscle	Knee OA group (1) median (IQR)	Old control group (2) median (IQR)	Young control group (3) median (IQR)	P value Kruskal Wallis	P value Pairwise comparisons
Baseline GM	4.225 (0.19) m/s	4.048 (0.27) m/s	4.013 (0.17) m/s	0.172	
Lift shank GM	4.164 m/s	4.099 (0.25) m/s	4.058 (0.26) m/s	0.621	
Double-leg stance GM	4.270 (0.027) m/s	4.227 (0.22) m/s	4.142 (0.23) m/s	0.190	
Single-leg stance GM	4.405 (0.28) m/s	4.390 (0.36) m/s	4.309 (0.26) m/s	0.489	
Baseline VL	4.235 (0.32) m/s	4.185 (0.21) m/s	4.004 (0.25) m/s	0.027*	(1) vs. (3) 0.016* (2) vs. (3) 0.020*
Lift shank VL	4.094 (0.26) m/s	4.097 (0.26) m/s	4.030 (0.15) m/s	0.502	
Double-leg stance VL	4.099 (0.30) m/s	4.230 (0.22) m/s	4.165 (0.56) m/s	0.351	
Single-leg stance VL	4.093 (0.26) m/s	4.309 (0.25) m/s	4.084 (0.25) m/s	0.259	

Abbreviations: GM, gastrocnemius medialis muscle, IQR, interquartile range; OA, osteoarthritis; VL, vastus lateralis muscle; vs., *versus*; *P < 0.05.

### 3.3 Ratios of longitudinal-to-transverse stiffness and *single-leg stance*-to-*baseline* stiffness

**FIGURE 5:**
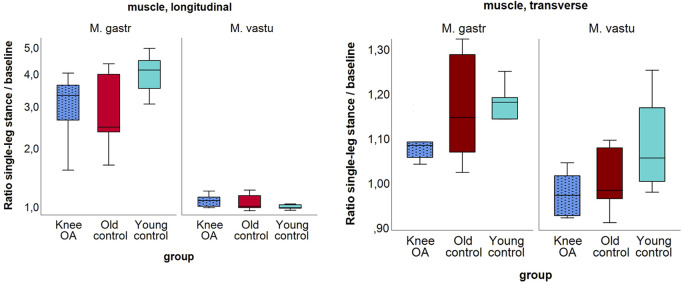
Ratio of shear wave velocity at single-leg stance relative to baseline of the longitudinal (left) and the transverse (right) shear wave velocity of the gastrocnemius medialis and vastus lateralis muscles per group; (left) in logarithmic scaling. Note the generally higher ratio for the gastrocnemius muscle due to higher longitudinal stiffness at stance.

In the *knee OA* group, the median ratio of longitudinal-to-transverse SWV was significantly lower for the gastrocnemius medialis muscle at *single leg stance*, P = 0.049, with tendencies toward a lower ratio in the OA group compared to the control groups in all but one measured condition (Table 6). Although not statistically significant (P = 0.051 for transverse SWV of the vastus lateralis muscle), the low *single-leg stance*-to-*baseline* ratio of transverse SWV suggests the least change in transverse muscle stiffness with activity in the *knee OA* group (Figure 5).

**TABLE 6:** Ratio of longitudinal relative to transverse SWV of the gastrocnemius medialis and vastus lateralis muscles per group at resting state and tasks of increasing activity.

Ratio long. SWV /transv. SWV M. Gastroc. Med. V. vastus lateralis	Baseline median (IQR)	Lift shank median (IQR)	Double-leg stance median (IQR)	Single-leg stance median (IQR)
Knee OA group	1.008 (0.05) 0.977 (0.05)	0.993 0.997 (0.08)	1.159 (0.44) 0.987 (0.10)	1.427 (0.64)* 1.054 (0.06)
Old control group	1.037 (0.07) 0.990 (0.04)	1.028 (0.05) 1.012 (0.04)	1.239 (0.48) 1.026 (0.10)	1.588 (0.59) 0.990 (0.09)
Young control group	1.009 (0.02) 1.011 (0.03)	1.008 (0.06) 1.011 (0.08)	1.314 (0.42) 0.996 (0.08)	1.775 (0.49) 1.000 (0.08)

*P < 0.05. Note the lowest median ratio in the knee OA group across all recording conditions except for single-leg stance, M. vastus lateralis.

### 3.4 Correlation of stiffness measures with patient reported outcomes

Longitudinal SWV demonstrated significant moderate to excellent correlations with the KOOS symptoms score (*double-leg stance*, *ρ* = 0.487 P = 0.047), the KOOS QoL score (*double-leg stance*, *ρ* = 0.724 P = 0.005), and with the Tampa Scale of Kinesiophobia (*baseline*, *ρ* = 0.668 P = 0.025). Transverse SWV was well to excellently correlated with the KOOS ADL score (*baseline ρ* = 0.812 P = 0.049; *double-leg stance ρ* = 0.679 P = 0.022), with the KOOS QoL score (*double-leg stance ρ* = 0.668 P = 0.025) and with the Tampa Scale of Kinesiophobia (*baseline ρ* = 0.856 P < 0.001; *lift shank ρ* = 0.748 P = 0.020) The consistently different signs of the correlations of longitudinal SWV with the KOOS pain score suggest separate consideration of the low activation conditions *baseline* and *lift shank versus* the standing tasks (Table 7). Inconsistencies across tasks and muscles request cautious interpretation of the correlation results.

**TABLE 7:** Spearman correlation coefficients of longitudinal and transverse shear wave velocity of the gastrocnemius medialis and vastus lateralis muscles in the knee OA group at resting state and tasks of increasing activity.

Longitudinal SWV at task	Muscle (n)	Perceived stiffness	KOOS pain	KOOS symptoms	KOOS ADL	KOOS sport	KOOS QoL	TSK
*Baseline*	Gastroc. (7)	−0.382	−0.393	0.704	0.577	−0.090	0.556	0.055
	Vast.lat. (11)	0.168	−0.137	−0.147	0.068	−0.189	0.257	0.668*
*Lift shank*	Gastroc. (7)	0.109	−0.393	0.334	0.631	0.324	0.482	0.164
	Vast.lat. (11)	−0.220	−0.384	0.078	−0.297	−0.028	0.028	0.371
*Double-leg stance*	Gastroc. (17)	−0.145	0.198	0.487*	0.281	0.277	0.220	−0.080
	Vast.lat. (13)	−0.431	0.480	0.385	0.482	0.382	0.724*	−0.061
*Single-leg stance*	Gastroc. (17)	−0.005	0.419	0.314	0.444	0.481	0.123	−0.248
	Vast.lat. (12)	−0.329	0.011	0.060	0.350	0.120	0.380	−0.214
Transverse SWV at task								
*Baseline*	Gastroc. (6)	0.232	−0.371	0.000	0.812*	0.464	0.030	0.232
	Vast.lat. (11)	0.150	−0.037	−0.189	0.123	−0.202	0.202	0.856**
*Lift shank*	Gastroc. (2)	n.a	n.a	n.a	n.a	n.a	n.a	n.a
	Vast.lat. (9)	0.094	0.134	−0.281	0.118	−0.294	0.118	0.748*
*Double-leg stance*	Gastroc. (11)	−0.065	0.460	0.484	0.679*	0.556	0.668*	−0.065
	Vast.lat. (6)	0.000	−0.029	0.200	−0.143	−0.143	−0.794	0.783
*Single-leg stance*	Gastroc. (16)	0.198	0.303	0.269	0.496	0.062	0.144	0.198
	Vast.lat. (8)	0.061	−0.252	−0.214	−0.238	−0.347	0.133	0.503

*P < 0.05; **P < 0.001. Abbreviations: ADL, activities of daily living; Gastroc., M. gastrocnemius medialis; KOOS, knee osteoarthritis outcome score; QoL, quality of life; SWV, shear wave velocity; TSK, Tampa scale of kinesiophobia; Vast.lat., M. vastus lateralis. Significant correlations are printed in bold font for easier recognition.

Considering all participants of the OA group irrespective the most painful muscles, perceived stiffness correlated significantly with age, *ρ* = 0.491 P < 0.001, BMI *ρ* = 0.310 P = 0.046, and all KOOS scores: Pain, *ρ* = −0.526 P < 0.001, ADL *ρ* = −0.469 P = 0.002, Sport *ρ* = −0.454 P = 0.003, and QoL *ρ* = −0.490 P < 0.001; but not with measured muscle stiffness.

## 4 Discussion

The current study results suggest lower longitudinal stiffness in the painful medial gastrocnemius and vastus lateralis muscles of individuals with knee OA during standing positions compared to age- and sex-matched controls. Conversely, results suggest higher muscle stiffness in the transverse plane, with significant differences in the vastus lateralis muscle at baseline. Across all groups, transverse muscle stiffness increased only marginally with activity, with the smallest increase observed in the knee OA group. Significant moderate to excellent correlations suggest meaningful relationships between both longitudinal and transverse shear wave velocity measurements, the KOOS subscores, and kinesiophobia, but not with the sensation of stiffness.

We identified no comparable studies specifically examining muscle stiffness in individuals with knee OA. Gökşen et al. (2021) used shear wave elastography to investigate stiffness in the rectus femoris and biceps femoris muscles in individuals with early knee OA compared to healthy individuals. They observed highly variable stiffness in both groups and muscles, with a trend toward higher median stiffness in the rectus femoris and lower median stiffness in the biceps femoris muscles in participants with knee OA. Thirty-one participants with early-stage knee OA were measured in a prone position with 30°–45° knee flexion. Several small measurements zones were manually positioned within the elastogram by an examiner; blinding was not reported (Gökşen et al., 2021). In contrast, Li et al. (2021) found significantly elevated stiffness in the hamstring muscles of 50 study participants with knee OA and symptoms of pain, stiffness, and heightened sensitivity in the posterior knee region. Apparently, stiffness measures were recorded in a prone position with an extended knee (2021). The measurement zone was larger than that used in the study by Gökşen et al., but similarly, blinding of the examiner was not reported. In both studies, the manual and maybe unblinded positioning of small measurement zones within muscles of heterogeneous stiffness may have introduced bias (Haueise et al., 2023).

A further study examined 79 participants aged 60–80 years without knee pain or known arthritis and found that increased passive longitudinal stiffness of the quadriceps femoris muscles (summed measures of the vastus lateralis, vastus medialis and rectus femoris muscles) was associated with a higher risk of developing knee OA within 12 months (Li et al., 2023). Measurements were recorded in a supine position with 60° of knee flexion using a computed extraction of stiffness from a large muscle region. The authors suggest that increased quadriceps muscle stiffness may contribute to a higher risk of developing knee OA (Li et al., 2023). We acknowledge the methodological strength of this study, which suggests increased longitudinal muscle stiffness precedes the onset of knee OA. The examined sample and the study question differ from the here presented work.

The aforementioned knee OA studies report inconsistent results and are not comparable to the present study in terms of design or measurement methods. Participants were not asked to identify their most painful muscles nor were functionally relevant weight-bearing conditions included. In the present study, we recorded elastography clips comprising 24–54 elastograms, over which stiffness measures over the full width of the transducer’s footprint were averaged, resulting in highly reliable and representative measurements. The extraction of muscle stiffness has been performed fully computed and thus blinded for group allocation. The current results align with previous work that found equal or lower longitudinal stiffness of muscles with chronic pain (Dieterich et al., 2020; Friede et al., 2020; Klauser et al., 2022; Wang et al., 2022). Longitudinal muscle stiffness relates to the contractile tension built up during muscle activation (Nordez and Hug, 2010; Hug et al., 2015). Thus, lower active longitudinal stiffness in painful muscles suggests reduced muscle activation around the painful arthritic knee joint.

In the past years, transverse muscle stiffness was increasingly measured. Studies focussed on methodological and reliability assessments in healthy participants (Gennisson et al., 2010; Eby et al., 2013; Miyamoto et al., 2015; Chino et al., 2017; Ruby et al., 2019; Vuorenmaa et al., 2022; Ngo et al., 2024). Additionally, a few diagnostic studies have employed transverse stiffness measurements (Nicholls et al., 2020; Kolb et al., 2021a; Kolb et al., 2021b). Most of these studies measured muscle stiffness in a relaxed state. Some studies included assessments under increasing active load in human volunteers (Gennisson et al., 2010) or passive load in specimen (Eby et al., 2013; Ngo et al., 2024). The existing literature confirms lower (Gennisson et al., 2010; Eby et al., 2013; Vuorenmaa et al., 2022; Ngo et al., 2024) or equal (Kolb et al., 2021b) transverse compared to longitudinal stiffness measures in healthy individuals and only marginal increases of transverse stiffness under loading. In systemic sclerosis (Kolb et al., 2021b) and in myositis (Kolb et al., 2021a), increased passive transverse muscle stiffness has been identified as biomarker for disease. Correspondingly, the current study suggests higher transverse stiffness in the knee OA group, with a significant difference observed in the vastus lateralis muscle at baseline. A clinical relevance of transverse stiffness measurements is underlined by the correlation results.

Longitudinal and transverse shear wave elastography measure the velocity of shear waves induced by focused acoustic beams. Consequently, shear wave elastography relies on shear wave propagation along echogenic structures that are organized approximately parallel to the transducer’s footprint. The transverse mechanical properties of muscles are primarily influenced by the connective tissue between muscle fibers - the extracellular matrix (Purslow, 2020; Wheatley, 2020). The extracellular matrix forms a complex network of proteins and fibers that envelop muscle fibers, anchoring them to facilitate efficient force transmission during contraction and movement (Ramaswamy et al., 2011; Marcucci et al., 2019; Sinha et al., 2020; Minato et al., 2021; Wohlgemuth et al., 2023; Spadoni et al., 2024). Additionally, it adapts to the mechanical conditions of the muscle (Jozsa et al., 1988; Järvinen et al., 2002; Kjaer et al., 2006; Hyldahl et al., 2015; Gihring et al., 2020). Thus, transverse muscle stiffness may be interpreted as the elasticity of the connective tissue between muscle fibers.

Compared to longitudinal measurements, transverse measures of muscle stiffness are technically challenging due to an increased difficulty in tracking shear waves (Eby et al., 2013). This is indicated by areas of void pixels where stiffness information is absent. Since shear wave tracking depends on echogenic structures along which shear waves propagate, the orientation of the intramuscular connective tissue is of high interest. Purslow described the endomysial network as a “planar feltwork of quasi-randomly orientated, wavy fibers” lying in a plane parallel to the muscle fiber surface (2020). Similarly, the perimysium, which ensheathes several muscle fiber bundles, has been described as a lattice of wavy collagen fiber bundles arranged at +55° and −55° angles relative to the muscle fiber direction (Purslow, 2010). A famous cross-sectional electron micrograph of the endomysial and perimysial networks (Purslow and Trotter, 1994; Purslow, 2020; Figure 1) suggests structures which probably enable shear wave propagation approximately perpendicular to the muscle fibers. Variations in the orientation of the intramuscular connective tissue may increase the difficulty of shear wave transmission and tracking, potentially resulting in lower quality elastograms. However, Miyamoto et al. (2015), Eby et al. (2013) and Gennisson et al. (2010) reported negligible stiffness differences in measurements angled up to 45° and 90° relative to muscle fiber direction suggesting that transverse stiffness measures are robust even when the measurement direction is not perfectly aligned with the echogenic structures.

In the present study, the low *single-leg stance*-to-*baseline* ratio of transverse stiffness in the *knee OA* group suggests the least stiffness change with high-level contraction compared to the control groups. Also, a trend toward higher transverse stiffness in the *knee OA* group was observed, both pointing to reduced elasticity of the between-fiber connections. During contraction, muscle deforms in three dimensions while maintaining constant volume (Ryan et al., 2020). Modelling studies suggest that limiting the radial expansion of a muscle, i.e., restricting transverse force, constrains longitudinal contraction (Gindre et al., 2013; Azizi et al., 2017; Ryan et al., 2020). Higher transverse muscle stiffness may therefore contribute to reduced muscle contraction levels. With the longitudinal-to-transverse stiffness ratio, we combined longitudinal and transverse stiffness measurements as a two-dimensional descriptor. In the OA group, the longitudinal-to-transverse ratio was lowest for both muscles across most tasks, suggesting reduced longitudinal tension relative to increased between-fiber stiffness. Interestingly, palpation and massage techniques are usually applied transversely to muscle fiber direction, implying an intuitive understanding of the importance of between-fiber elasticity.

The current work has important limitations. Most notably, the selection of the most painful muscles for assessment resulted in four different muscles being evaluated, dividing the sample into subgroups and reducing the statistical power of the study. Measuring the most painful muscles was mandatory for answering the research question and represented the clinically most relevant approach. More data are needed to confirm the observed tendencies. The current work enables sample size calculations for larger studies. Further, the sensation of stiffness in OA is typically reported when starting movement. Probably, we diminished group differences by the standardized warm-up procedure prior to data acquisition. We standardized prior activity because it influences muscle length (Proske and Gandevia, 2012), and therefore likely also muscle tension and stiffness. Moreover, the technical challenges associated with transverse stiffness measurements resulted in fewer data being included in the statistical analysis. This affected predominantly the transverse measurements of the vastus lateralis muscle. Only the use of elastography clips comprising 24–54 elastograms, from which measurements were averaged, enabled the high data inclusion and good to excellent reliability. In light of these limitations, the present findings should be interpreted with caution. Nonetheless, the observed trends, statistically significant differences and correlations suggest that the inclusion of transverse muscle stiffness measurements may contribute to a more comprehensive understanding of muscle mechanical properties and hold potential clinical relevance. Future studies should expand the sample size to further validate the current observations. This study did not include an assessment of biomechanical factors that may also influence muscle stiffness, such as lower limb alignment. Future research should consider incorporating biomechanical measurements to evaluate their potential impact on muscle stiffness.

## 5 Conclusion

In individuals with knee osteoarthritis, the stiffness of painful muscles was not elevated along the muscle fibers; rather, it was reduced during weight-bearing conditions. The inclusion of transverse muscle stiffness measurements appears to offer additional clinically relevant insights and may contribute to a more comprehensive assessment of muscle mechanics in this population. The sensation of stiffness did not align with measured stiffness.

## Data Availability

The datasets presented in this study can be found in online repositories. The names of the repository/repositories and accession number(s) can be found below: The raw data analyzed for this study can be found in ZENODO, doi 10.5281/zenodo.15025467 and 10.5281/zenodo.15025478.
